# Is quality of life post cardiac surgery overestimated?

**DOI:** 10.1186/1477-7525-12-62

**Published:** 2014-04-29

**Authors:** Luc Noyez

**Affiliations:** 1 Heart Center, Radboud University Nijmegen Medical Center, Department of Cardio-Thoracic Surgery – 677, PO Box 9101, Nijmegen 6500 HB, Netherlands

**Keywords:** Quality of life, Cardiac surgery, Elderly, Risk

## Abstract

**Background:**

Quality of Life (QoL) studies concerns the difference in QoL between the baseline and the post-surgical assessment. Many such studies, however, suffer from incomplete QoL-data with regard to patients with a proven survival - the drop-outs. Our hypothesis is that patients with a low preoperative QoL, high operative risk and older age are at higher risk for drop-out, which may result in a biased conclusion.

**Methods:**

This study includes 1675 patients, all of whom were operated between July 1, 2009 and July 1,2012 and have a proven one-year survival, as well as a complete preoperative EuroQoL registration (EQ-5D and EQ-VAS). Based on the calculated 30 and 70 percentiles of age, EuroSCORE risk, and EQ-5D and EQ-VAS values, the group was split into three different subgroups. We studied whether (1) there was a correlation between age, risk, preoperative QoL and postoperative QoL and (2) if the drop-outs were correlated to age, risk and preoperative QoL.

**Results:**

There is a statistically significant correlation between postoperative QoL and both age (p = 0.029) and risk (p = 0.002). Both relations have a negative Pearson’s r. There is also a statistically significant (p = 0.0001) correlation between pre- and postoperative QoL, now with a positive Pearson’s r. The percentage of drop-outs increases in a statistically significant manner with an increased risk (p = 0.001), older age (p = 0.001) and a low preoperative QoL (EQ-5D, p = 0.001 and EQ-VAS, p = 0.003).

**Conclusion:**

We conclude that QoL post cardiac surgery is overestimated, certainly for older, high risk patients and patients with a low preoperative QoL.

## Background

Over the past years, Quality of Life (QoL) has become an increasingly important aspect in medicine, social sciences and health care. Even evidence based medicine now attributes importance not only to the direct physical impact of the disease and the results of treatment, but also to a patient’s QoL. In the recently updated ESC/EACTS guidelines on the management of valvular heart disease (version 2012) it is noted that ‘quality of life issues’ should be taken into account when deciding on the type of valve [1]. Several papers have already dealt with the evaluation of QoL post cardiac surgery, mostly with regard to elderly patients [2-5]. Here, QoL research concerns an evaluation of preoperative versus postoperative QoL. In a previous paper we have already called for consideration of data about demographics, co-morbidity and cardiac risk of patients who are excluded in QoL evaluation studies [6]. In this current study we focus on yet another group: patients with preoperative QoL information and a proven survival, but with a lack of or incomplete postoperative QoL information at the moment of the evaluation - the “drop-outs”. Our hypothesis is that the identity of these drop-outs is related to age, operative risk and preoperative QoL.

The aim of the present study, then, is to evaluate whether the profile of the drop-out patients can bias the results of the QoL evaluation at one year post cardiac surgery.

### Patients and methods

#### Patients

**Figure 1:**
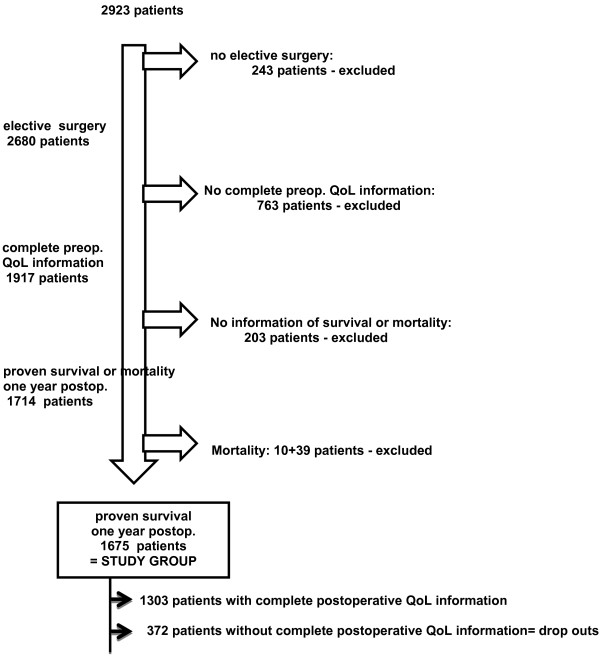
**Flowchart, illustrating the number of excluded and included patients.** Preop. = preoperative, postop. = postoperative.

From our cardiac surgery database - CORRAD, a database that stores pre-, per-, and post-operative data as well as follow-up data from all adult patients undergoing cardiac surgery at the Radboud University Nijmegen Medical Center (UMCN) – we identified, after exclusion of patients with a transcatheter aortic valve implantation, 2923 patients who underwent cardiac surgery between July 1, 2009 and July 1, 2012. (Figure 1. Flowchart) 2680 of these patients (91.4%) underwent elective surgery - the only type of surgery included in this QoL study. Complete preoperative QoL information was available for 1917 elective patients (71.5%) and at one year postoperative, there is proven survival or mortality information for 1714 (89%) of them. After exclusion of 49 patients who died during the first postoperative year - 10 patients died postoperative in hospital, 39 during the first postoperative year - we have a proven one year survival of 1675/1714 patients (97.7%). Complete postoperative QoL information is available for only 1303 (77.8%) of these patients, however, thus defining a drop-out group of 372 patients (22.2%). Our study population is comprised of the 1675 patients with complete preoperative QoL information and a proven one year survival.

#### Quality of life and operative risk stratification

To assess QoL both components of the EuroQOL instrument (EQ-5D and EQ-VAS) were used [7]. This is a validated standardized generic instrument to measure QOL. The EQ-5D consists of five domains of health (Mobility, Self-Care, Usual Activities, Pain/Discomfort, and Anxiety/Depression), each of which is divided into three levels: no problems (i), some or moderate problems (ii), and extreme problems (iii). Based on the response to this classification, a single index value is estimated using a general population-based algorithm [8]. For the EQ-VAS, patients estimate their own health on a visual analogue scale ranging from 0 to 100, with 0 being the worst possible health state and 100 being the best. The EQ-5D index can be regarded as a societal-based composite global QoL measure, whereas the EQ-VAS is a direct global QoL assessment from the patient’s perspective. Patients were asked to individually complete the preoperative QoL questionnaire on the day before surgery. Thus, only patients undergoing elective surgery were included in our QoL studies, and only patients with complete preoperative QoL information (both EQ-5D and EQ-VAS) were retained in this particular study.

The initial-logistic-EuroSCORE was used for risk stratification [9]. The EuroSCORE is a simple, objective system for assessing heart surgery and derived from an international European database. The calculated EuroSCORE (http://www.euroscore.org) is based on patient-, cardiac- and operation-related factors (Table 1).

**Table 1:** Risk factors in the EuroSCORE

**Risk**	**Risk factor**
Patient-related	
	Age
	Female gender
	Chronic pulmonary disease
	Extracardiac arteriopathy
	Neurological dysfunction
	Previous cardiac surgery
	Serum creatinine
	Active endocarditis
	Critical preoperative state
Cardiac-related	
	Unstable angina
	Left ventricular dysfunction
	Recent myocardial infarction
	Pulmonary hypertension
Operation-related	
	Emergency
	Other than isolated coronary artery bypass grafting
	Surgery on thoracic aorta
	Postinfarct septal rupture

#### Follow-up

The follow-up data results from our yearly follow-up, a written survey sent directly to the patients. This survey includes the same QoL questionnaire as given preoperatively. Participation in the survey is voluntary - no patients in this study are contacted by phone or received a second mailing in this study. The registration of data in the CORRAD database and the use of this information for research have been approved by the local ethical and research council of the Radboud University Nijmegen [10].

## Method

Based on the 30 and 70 percentile of age, logistic EuroSCORE, ED-5D and EQ-VAS, the group of 1675 patients with complete preoperative QoL information is divided into three groups. A possible relation between age, EuroSCORE risk, preoperative QoL (EQ-5D and EQ-VAS) and the one year postoperative QoL (EQ-5D and EQ-VAS) is analyzed in the group of 1303 patients who provided complete postoperative QoL information. The impact of age, risk, and preoperative QoL on the drop out of patients is studied by analyzing the association of the three groups based on percentiles.

### Statistical analysis

Statistical analyses were performed using IBM SPSS statistics 20, Chicago, IL, USA. Characteristics of patients are presented as percentage for dichotome variables and as mean ± standard deviation and range for numerical variables. Differences in percentages were tested with the chi-square test and numerical variables were tested with the t-test or Mann–Whitney test when appropriate. Student’s t-test are performed to examine the mean differences and a Pearson’s r correlation test to measure how variables are related. If there is no relationship the correlation coefficient is 0, a perfect relationship results in a value of 1. A value >0.76 means a strong, 0.51-0.75 a medium and under 0.50 a low correlation. To investigate the dropout a Chi-squared test was used. Statistical significance is assumed at p ≤ 0.05.

### Study endpoints

There are two major endpoints: (1) Is there a correlation between preoperative age, risk, QoL and postoperative QoL? (2) Is there an association between preoperative age, risk, QoL and the number of drop out patients?

## Results

The group of 1675 patients has a mean age of 66.9 ± 10.3 (18–93) years, a logistic EuroSCORE risk of 4.5 ± 4.2 (0.88-51.13), and 451 (27.3%) are women. 1054 of the patients (62.9%) underwent an isolated CABG, 401 (23.9%) an aortic valve replacement, whether or not in combination with a CABG, 96 (5.8%) mitral valve surgery, whether or not in combination with a CABG and 124 (7.4%) another type of adult cardiac surgery. Preoperative EQ-5D was 0.72 ± 0.26 (−30 -1.00) and EQ-VAS was 63.3 ± 21,0 (0–100). This group of 1675 patients is, based on the thirtieth and seventieth percentile of age, EuroSCORE, EQ-5d and EQ-VAS divided in three groups (Table 2).

**Table 2:** **Partition of the total study-population (N = 1675) based on the 30**^**th **^**and 70**^**th **^**percentile for age, EQ-5D, EQ-VAS and logistic EuroSCORE**

	**<30**^**th **^**percentile, N**	**≥30**^**th **^**N ≤ 70**^**th**^	**> 70**^**th **^**percentile, N**
Age (years)	< 62, N:499	N = 676	>73, N = 500
EQ-5D	<0.72, N = 480	N = 549	>0.84, N = 646
EQ-VAS	<50, N = 311	N = 787	>75, N = 577
EuroSCORE risk	< 1.8, N = 500	N = 681	>4.6, N = 494

Table 3 shows the correlation between age, logistic EuroSCORE risk, preoperative EQ-5D, preoperative EQ-VAS and postoperative EQ-5D and EQ-VAS. There is no statistically significant correlation between age and post EQ-5D (p = 0.277), but there is between age and post EQ-VAS (p = 0.003). The Pearson’s r here is −0.082, meaning that a higher age correlates with a lower postoperative VAS. The logistic EuroSCORE has a statistically significant correlation with both the postoperative EQ-5D (p = 0.021) and VAS (p = 0.006). The Pearson’s correlation coefficient is −0.064 for EQ-5D and −0.137 for EQ-VAS, indicating that a higher logistic EuroSCORE risk correlates with lower postoperative EQ-5D and VAS, a so-called negative correlation. There is a statistically significant correlation between both the pre- and postoperative VAS (p = 0.001) and the pre- and postoperative ED-5D (p = 0.001). The Pearson’s correlation coefficient in these cases is 0.285 and 0.368, respectively, indicating that a higher preoperative value correlates with a higher postoperative value and inverse, a so-called positive correlation.

**Table 3:** Correlation between age, Logistic EuroSCORE risk, preoperative EQ-5D , preoperative EQ-VAS and postoperative EQ-5D and postoperative EQ-VAS in 1303 patients

**Studied correlation**	**Pearson’s r**	**p-value**
Age and postoperative EQ-VAS	−0.082	0.003
Age and postoperative EQ-5D	−0.030	0.277
EuroSCORE and postoperative EQ-VAS	−0.137	0.001
EuroSCORE and postoperative EQ-5D	−0.064	0.021
Preoperative and postoperative EQ-VAS	0.285	0.001
Preoperative and postoperative EQ-5D	0.368	0.001

The 372 drop outs are statisically significant older (p = 0.001), at higher risk (p = 0.001)and have a statistically significant lower QoL, EQ-5D (p = 0.001), EQ-VAS (p = 0.001) than the group of 1303 patients with complete pre an postoperative QoL information (Table 4) Table 5 shows that there are statistically significant higher percentages of drop-outs related with higher age (p = 0.001), higher EuroSCORE risk (p = 0.001), lower EQ-5D (p = 0.001) and lower EQ-VAS (p = 0.003).

**Table 4:** Difference between age, Logistic EuroSCORE risk, preoperative EQ-5D , preoperative EQ-VAS between the 372 drop outs and the 1303 patients with complete pre- and postoperative QoL information

**Variable**	**Drop outs**	**Complete**	**p-value**
	**N = 372**	**N = 1303**	
Age	68.7 ± 11.1 (18–86)	66.1 ± 10.0 (25–93)	0.001
EuroSCORE	4.1 ± 5.4 (0.88-42.5)	3.8 ± 3.9 (0.88-51.1)	0.001
EQ-VAS	59.5 ± 20.7 (0–100)	64.3 ± 21.1 (0–100)	0.001
EQ-5D	0.65 ± 0.30 (−0.26-1.00)	0.74 ± 0.24 (−0.30-1.00)	0.001

**Table 5:** Evaluation of the percentage of drop out in association with age, logistic EuroSCORE and preoperative QoL - EQ-5D and EQ-VAS

	**Total**	**Drop out**	**Complete**	**p-value**
	**N = 1675**	**N = 372 (%)**	**N = 1303 (%)**	
Age (years)				0.001
<62	499	89 (17,8)	410 (82.2)	
≥ 62 and ≤73	676	136 (20.1)	540 (79.9)	
>73	500	147 (29.4)	353 (70.6)	
EuroSCORE				0.001
< 1.8	500	63 (12.6)	437 (87.4)	
≥ 1.8 and ≤4.6	681	145 (21.3)	536 (78.7)	
>4.6	494	164 (33.2)	330 (66.8)	
EQ-VAS				0.003
<50	311	86 (27.7)	225 (72.3)	
≥ 50 and ≤75	787	182 (23.1)	605 (76.9)	
>75	577	104 (18.0)	473 (82.0)	
EQ-5D				0.001
<0.72	480	141 (29.4)	339 (70.6)	
≥ 0.72 and ≤0.84	549	111 (20.2)	438 (79.8)	
>0.84	646	120 (18.6)	526 (81.4)	

## Discussion

The evaluation of QoL post cardiac surgery involves a comparison of preoperative QoL to postoperative QoL. Each study has inclusion and exclusion criteria and some patients simply refuse to participate in a study. A number of patients die postoperatively, before the moment of QoL evaluation. Moreover, at the moment of QoL evaluation some patients are lost for follow-up, leaving us without any information about their survival or QoL. There are also patients with a proven survival, but with no or incomplete postoperative QoL information, a group we have called the “drop-outs”. In our previous paper we already discussed the importance of providing information about demographics, co-morbidity and cardiac risk of patients who were excluded or dropped out before generalization of the final QoL results [6]. In this paper, however, we focus on the particular impact of drop-outs on the results of the QoL evaluation. We have thus included only patients with complete preoperative QoL information and a proven one-year survival. These are the only patients from whom we can expect postoperative (one year) QoL information.

### Patients

Of 2680 elective patients, 1917 (71.5%) were enrolled in the study. It is comparable with the 71.4% inclusion in the study of Rumsfeld and much higher than a recent published study by Bramer et al., who had only had only about 40% of the patients with pre- and postoperative QoL information [5,11]. However lower than in a previous study where we had roughly 75% inclusion and the recent study of KH Gjeilo et al., which had circa 91% inclusion [4,12]. It is difficult to compare these percentages of inclusion, though, since several publications make no mention of such data and the method and criteria of inclusion and exclusion are also determining [6]. It is important to note that, in our study, the registration of preoperative QoL is an integral part of the admission procedure. This sets our study apart from those in which selected patients are individually invited to participate and increases the inclusion percentage.

During the first postoperative year 49 patients died, whereas there is proven one-year survival of 1675 patients. We have, in other words, a one-year follow-up of 89% of all patients with complete preoperative QoL information. This information is the result of our routinely performed follow-up as described in previous papers [10,13]. Not only have we registered death but also proven survival, because we must be sure of survival in order to expect some –one-year postoperative –QoL information. Complete postoperative QoL information is available for 1303/1675 patients (77.8%); the remaining 372 patients (22.2%) were identified as drop-outs. Circa half of these drop-outs were due to incomplete QoL information. The percentage of drop-outs may thus be decreased by contacting patients by telephone and completing the QoL questionnaire together with an interviewer. It is nevertheless remarkable that incomplete QoL information is seldom noted as an exclusion criteria in QoL studies.

### Correlation between age, preoperative risk , preoperative QoL and postoperative QoL

Table 2 shows no statistically significant correlation between age and EQ-5D, but a statistically significant, though negative, correlation with the EQ-VAS. A decrease in QoL with older age is known, and particular due to the physical dimensions of the QoL [2]. It is probable that the correlation did not reach statistical significance for the EQ-5D for exactly this reason, as the EQ-5D also considers other aspects. That it does reach statistical significance for the EQ-VAS can be attributed to the latter’s reflection of subjective expectations. Older patients are more disappointed when there is no more increase of QoL postoperative.

The preoperative risk correlates negatively but statistically significant with EQ-5D and EQ-VAS. This can be explained by the composition of risk on the basis of several co-morbidity variables such as gender, age, arterial vascular disease, neurological disease, lung disease and renal failure. These variables have their own influence on QoL, especially on the physical dimension. Everyone knows of patients with a good result of their CABG who nevertheless complain of an inferior QoL due to their arterial vascular disease. We have discussed this complex relation, especially for physical activity post CABG, in a previous article [14].

There is a statistically significant and positive correlation between preoperative and postoperative QoL, for both the EQ-5D and the EQ-VAS. It is known that the increase in QoL postoperatively is dependent on the preoperative QoL. Most studies focus on the fact that patients with a low preoperative QoL are most likely to have an increase in QoL, while patients with a high preoperative QoL are more likely to experience a decrease [5,15]. The same studies, however, as well as others, confirm an average improvement in QoL following cardiac surgery [2-5,13,15-17]. Patients starting with a high preoperative QoL, in other words, also tend to have a higher QoL postoperatively.

That our correlation values are low - - confirms again the complexity of QoL. There is no one to one correlation between the studied variables.

### Relation between dropout an preoperative age, risk and QoL

Table 3 shows that the percentage of drop-outs is statistically significantly higher with older age (p = 0.001), higher risk (p = 0.001) and lower preoperative QoL, in terms of both EQ-5D (p = 0.003) and EQ-VAS (p = 0.001). This means that the group of patients with both preoperative *and* postoperative QoL information is not only younger, but also at lower risk and has a better QoL to start with than the total group. This seems in contrast with our recent study concerning the importance of follow-up post cardiac surgery, in which we concluded that during the first follow-up year the patients lost for follow-up were younger and at lower risk [13]. Yet, an important difference here, is that the previous study focused only on data regarding survival and mortality, whereas the present study takes the availability of complete QoL data (pre- and postoperative) as an important inclusion criteria.

### Impact on the results of QoL evaluation

On the one hand, there are a statistically significant negative correlation between age, operative risk and postoperative QoL and a statistically significant positive correlation between good preoperative QoL and postoperative QoL. On the other hand, the fact that the drop-outs are of a statistically significant higher age, at higher risk and have a lower preoperative QoL leads us to conclude that the evaluations of QoL based on the relation between preoperative and postoperative QoL are made only on the basis of “the best” patients in the study and thus that such conclusions overestimate the postoperative QoL of the total group. Interesting here would be to identify events as severe illness, stroke, responsible for a number of the drop outs.

In addition, it points out that the missing QoL information is not at random (MNAR), which in turn has consequences for the eventual imputation of these data [17,18].

Of course, this study focuses only on a single institution and, as we’ve already discussed, the percentage of drop-outs could be decreased by a specific set–up of the study, by the individual invitation of patients to participate in or by individual patient contact postoperative by phone, or second mailing. Or, as in the study of van Geldorp et al., who invited patients for a specific echocardiogram according to the study protocol before inclusion [19]. An inclusion criteria, as this, results already in a declined participation of older and sicker patients and bias then the study. Another point is that only age, operative risk and preoperative QoL are included in the current analysis. Variables as type of operation, gender, frailty, physical activity, diabetes could also been selected.

## Conclusion

In conclusion, studies evaluating QoL post cardiac surgery should not only describe the group of excluded patients [6] but should also take into account that the so-called drop-outs may be older, at higher operative risk and have a lower preoperative QoL, three aspects that correlate with a lower postoperative QoL, and that the study’s results may thus be based on the best patients only and hence overestimate QoL post cardiac surgery for the total population.

## Competing interests

The author declares that he has no competing interests.
